# Welfare in Factories

**Published:** 1920-06-26

**Authors:** 


### THE PUBLIC WELL-BEING.

### Welfare in Factories.

### Care of Mothers, Babies, and Juveniles.

In a pamphlet recently issued by the Ministry of Labour it is stated that, regarding the question of health of juvenile workers, examination of conditions as found in over two thousand of the most important factories of the country has shown that "practically no attention is given to health conditions." This is a startling and serious admission which shows how necessary are the provisions of the new Education Act, though they may not prove complete. In hundreds of factories there is need of proper messing arrangements, better sanitary and washing accommodation, provision for recreation and suchlike, all whereof are closely connected with health and fitness. It is some satisfaction to find that the Ministry of Labour has a scientific eye over this important subject of industrial welfare, and in time we may expect reforms.

Some very interesting facts on welfare work were presented to the International Cotton Congress at Zurich, where it was held that great benefits would result if Governments allowed the cost of welfare work to be a trade charge. The discussion was opened by Sir E. Tootal Broadhurst, and it shed some light on the feminist contention that work which is not good for women is equally harmful for men. Sir E. Tootal Broadhurst said the increased extent to which women were employed in industry, especially on engineering work, established welfare work as an indispensable factor of health, efficiency, and production. The advantages of the work were not easily recorded by statistics, for they were more of mind and heart than matter, and their value was immeasurable. On personal inquiry of employers who had welfare schemes at work, one had never come across indifference. There was consistent enthusiasm. Records showed that under such schemes workers were happier and healthier, and there was definitely improved time-keeping. In July 1914, textile trades employed two-fifths of the total number of women employed in industry, and four-sevenths of the total number of textile operatives were women. In its relation to management, there should be no doubt of the necessity for welfare work, for, with the majority of women workers, where every grade of management is in the hands of men, there is abundant reason in the physical, temperamental, and moral affairs of women for the appointment of women welfare workers.

#### Rules in Other Countries.

The congress in general was strongly in favour of welfare work, and it was stated that Switzerland already allows the cost to be made a trade charge. A questionnaire had been sent to the associations represented in the International Federation on whether cotton-spinning is an unhealthy occupation, and the replies—though in many respects technical in character—gave valuable information. In Czecho-Slovakia, one of the new young States, children up to fourteen are not allowed to be employed in industrial concerns, and to those up to the age of eighteen the employer is compelled to allow the necessary time for attendance at continuation schools. Denmark has some sensible regulations. Concerns employing twenty-five women or above must be provided with a separate room where the babies of the women employed can be breast fed. The room must be heated during the cold season. Clothes not used while workpeople are working must, as far as possible, be kept away from any room in which work is carried on. If such articles of dress are kept in any corridor or in special rooms, each worker must have his own division for his clothes. If clothes are kept in the room where work is performed, or where meals are to be taken, the room must be provided with wardrobes containing divisions for the clothes of each worker. In the workroom or near it there must be well-cleaned arrangements for washing; not less than one washing arrangement for five workpeople. Each person should use a separate towel, and a clean one should be provided every week. Before commencing any meal and before leaving the workplace the workpeople should wash their hands and faces. Overalls should be changed at least once a fortnight.

#### Nursing-room for Babies.

In Holland notices must be affixed explaining first aid in case of accidents. Where more than fifty persons are employed there must be at least one person familiar with first aid in case of accidents. No children under fourteen years of age may be employed. In Italy a nursing-room for the feeding of infants is compulsory in all factories where at least fifty women are employed. Children up to fifteen, and women up to twenty-one, are not allowed to undertake manual labour of an industrial character, unless they obtain a medical certificate which sets out that they are healthy and suitable for the work intended. They must present themselves for examination every time they undertake a different class of work, or in cases where a factory inspector considers that the health of a female worker would suffer from the continuation of the work at which she may be engaged. Children and women under twenty-one must not be employed in rooms where certain processes injurious to health are carried on.

At Norwegian mills women are not permitted to work until six weeks after confinement. In Spain the minimum period is a month, or it may be six weeks, and the employer must keep the woman's place open for her. One who is entering upon the eighth month of child-bearing may ask to be released from work, and her place must be kept open for her. Women who nurse their own children must be given one hour per day within the working time to enable them to feed their infants. The

**THE PUBLIC WELL-BEING**—(*continued*).

hour must be divided into two periods of thirty minutes, one in the morning and one in the afternoon, and it is not permissible to take from her day's wage the time used for this purpose. Children of both sexes up to sixteen and women under age are prohibited from working in certain cases considered injurious to health.

